# Supplementary material for: Health Priorities and Participation in Peer-Led Active Rehabilitation Camps Among Persons with Spinal Cord Injury: A Prospective Cohort Study
Source: J Clin Med. 2025 Dec 25;15(1):176. doi: 10.3390/jcm15010176 (PMC12787264; doi:10.3390/jcm15010176)
Supplement: Supplementary file 1 [file jcm-15-00176-s001.zip › jcm-4004630-supplementary.pdf]

Supplementary Table S1

No	Fidelity criteria	Mean	Min	Max	SD	Criterion meeting
1a	Peer mentors with a ratio of at least one peer mentor for every five participants; (ratio peer mentors : participants)	1:3	1:2	1:3		16/16
1b	Peer mentors leading a minimum of 80% of all structured sessions; (% of peer mentors)	80%	46%	100%		15/16
2	Trained non-disabled assistants	8.6	4	18	3.4	16/16
3	ADL and wheelchair skills training provided by peer mentors on average at least 90 minutes daily; (minutes)	149	108	180	19,8	16/16
4	Physical training, sports and therapeutic recreation activities incorporated in the schedule on average at least 90 minutes daily; (minutes)	151	99	171	18	16/16
5	Formal educational sessions (e.g. on bowel and bladder management; sexual function) on average at least 30 minutes daily; (minutes)	87.3	54	180	27.9	16/16
6	Training environment in the community					16/16
7	Specific admission criteria (i.e. being free of severe medical complications; being able to push a manual wheelchair on an even surface; being able to follow instructions; not having problems with concentration, memory and orientation);					16/16
	- At least half of the participants having a SCI; (n)					16/16
	- Participants with a SCI; (n)	8.9	3	15	3.6	
	- At least half of the peer mentors having a SCI; (n)					16/16
	- Peer mentors with a SCI; (n)	4	2	5	0.8	16/16
8	A goal-setting process is part of the programme;					16/16
9	Peer mentors having received formal training about AR;	100%	100%	100%	0	16/16
10	Duration of the AR programmes between 7-15 days; (days)	10	10	10	0	16/16
	- Included participants attending at least 3/4 of the actual programme duration; (n)	8,2	3	15	3,2	16/16

Supplementary Table S2

Independent variables	Dependent variables					
	Mobility index [A]				Bowel and Bladder index [A]	
	T2-T1		T3-T1		T2-T1	
	**Test result**	**P value**	**Test result**	**P value**	**Test result**	**P value**
Age (years)	0.12[a]	0.264	0.18[a]	0.088	0.08[a]	0.405
Time since injury (years)	–0.25[a]	**0.017**	–0.19[a]	0.079	–0.08[a]	0.424
Sex (1* – male, 2 – female)	0.81[c]	0.423	–0.12[b]	0.908	0.80[c]	0.427
Marital status (1* – single, 2 – living in relationship)	0.64[c]	0.524	0.15[b]	0.884	0.98[c]	0.332
AR camps attendance (1* – first time, 2 – more than once)	3.29[c]	**0.002**	1.83[c]	0.071	0.22[b]	0.827
Level of SCI (1* – paraplegia, 2 – tetraplegia)	0.70[c]	0.488	1.29[c]	0.202	0.88[b]	0.380
Completeness of SCI (1* – complete, 2 – incomplete)	–0.78[c]	0.443	–0.19[b]	0.855	–0.56[b]	0.578
Cause of SCI (1* – non-traumatic, 2 – traumatic)	2.21[c]	**0.030**	0.83[b]	0.411	0.65[c]	0.519

Note: Bold – significant result at $p<0.05$; 1* – reference option; [a] Spearman's rank correlation coefficients (rs); [b] Mann-Whitney test (Z); [c] T-test; [A] All participants; [T] Participants with tetraplegia only; T1, T2, T3 – time of measurement

Supplementary Table S2 continuation

Independent variables	Dependent variables							
	Sexuality index [A]				Hand and Arm index [T]			
	T2-T1		T3-T1		T2-T1		T3-T1	
	Test result	P value	Test result	P value	Test result	P value	Test result	P value
Age (years)	0.26[a]	**0.012**	0.18[a]	0.077	–0.20[a]	0.200	0.01[a]	0.974
Time since injury (years)	–0.09[a]	0.380	–0.29[a]	**0.005**	–0.19[a]	0.222	–0.30[a]	0.055
Sex (1* – male, 2 – female)	1.35[b]	0.179	1.78[c]	0.080	–2.23[b]	**0.027**	–0.86[c]	0.400
Marital status (1* – single, 2 – living in relationship)	0.98[c]	0.334	0.26[c]	0.803	0.00[b]	1.000	–1.33[c]	0.194
AR camps attendance (1* – first time, 2 – more than once)	0.12[c]	0.908	1.51[c]	0.137	–0.89[b]	0.378	–1.29[c]	0.208
Level of SCI (1* – paraplegia, 2 – tetraplegia)	0.25[c]	0.804	–0.29[c]	0.778	N/A		N/A	
Completeness of SCI (1* – complete, 2 – incomplete)	0.84[b]	0.404	–1.10[b]	0.273	0.30[b]	0.768	1.29[c]	0.205
Cause of SCI (1* – non-traumatic, 2 – traumatic)	1.42[c]	0.161	0.89[c]	0.380	0.02[b]	0.988	1.38[c]	0.176

Note: Bold – significant result at $p<0.05$; 1* – reference option; [a] Spearman's rank correlation coefficients (rs); [b] Mann-Whitney test (Z); [c] T-test; [A] All participants; [T] Participants with tetraplegia only; T1, T2, T3 – time of measurement

STROBE Statement—Checklist of items that should be included in reports of ***cohort studies***

	Item No	Recommendation	Page No
**Title and abstract**	1	(*a*) Indicate the study's design with a commonly used term in the title or the abstract<br>(*b*) Provide in the abstract an informative and balanced summary of what was done and what was found	1-2
**Introduction**			
Background/rationale	2	Explain the scientific background and rationale for the investigation being reported	4-5
Objectives	3	State specific objectives, including any prespecified hypotheses	5
**Methods**			
Study design	4	Present key elements of study design early in the paper	5
Setting	5	Describe the setting, locations, and relevant dates, including periods of recruitment, exposure, follow-up, and data collection	5
Participants	6	(*a*) Give the eligibility criteria, and the sources and methods of selection of participants. Describe methods of follow-up<br>(*b*) For matched studies, give matching criteria and number of exposed and unexposed	5
Variables	7	Clearly define all outcomes, exposures, predictors, potential confounders, and effect modifiers. Give diagnostic criteria, if applicable	7-10
Data sources/ measurement	8*	For each variable of interest, give sources of data and details of methods of assessment (measurement). Describe comparability of assessment methods if there is more than one group	7-10
Bias	9	Describe any efforts to address potential sources of bias	11
Study size	10	Explain how the study size was arrived at	10 (ref protocol)

Quantitative variables	11	Explain how quantitative variables were handled in the analyses. If applicable, describe which groupings were chosen and why	10-11
Statistical methods	12	(*a*) Describe all statistical methods, including those used to control for confounding<br><br>(*b*) Describe any methods used to examine subgroups and interactions<br>(*c*) Explain how missing data were addressed<br>(*d*) If applicable, explain how loss to follow-up was addressed<br>(*e*) Describe any sensitivity analyses	10-11 (ref protocol)
**Results**			
Participants	13*	(a) Report numbers of individuals at each stage of study—eg numbers potentially eligible, examined for eligibility, confirmed eligible, included in the study, completing follow-up, and analysed<br>(b) Give reasons for non-participation at each stage<br>(c) Consider use of a flow diagram	12 & Figure S1
Descriptive data	14*	(a) Give characteristics of study participants (eg demographic, clinical, social) and information on exposures and potential confounders<br>(b) Indicate number of participants with missing data for each variable of interest<br>(c) Summarise follow-up time (eg, average and total amount)	12 & Tables
Outcome data	15*	Report numbers of outcome events or summary measures over time	12-13 & Tables

Main results	16	(*a*) Give unadjusted estimates and, if applicable, confounder-adjusted estimates and their precision (eg, 95% confidence interval). Make clear which confounders were adjusted for and why they were included<br>(*b*) Report category boundaries when continuous variables were categorized	12-13 & Figures

		(*c*) If relevant, consider translating estimates of relative risk into absolute risk for a meaningful time period	
Other analyses	17	Report other analyses done—eg analyses of subgroups and interactions, and sensitivity analyses	13-14
**Discussion**			
Key results	18	Summarise key results with reference to study objectives	14
Limitations	19	Discuss limitations of the study, taking into account sources of potential bias or imprecision. Discuss both direction and magnitude of any potential bias	16-17
Interpretation	20	Give a cautious overall interpretation of results considering objectives, limitations, multiplicity of analyses, results from similar studies, and other relevant evidence	14-17
Generalisability	21	Discuss the generalisability (external validity) of the study results	17
**Other information**			
Funding	22	Give the source of funding and the role of the funders for the present study and, if applicable, for the original study on which the present article is based	18

* Give information separately for exposed and unexposed groups.

Note: An Explanation and Elaboration article discusses each checklist item and gives methodological background and published examples of transparent reporting. The STROBE checklist is best used in conjunction with this article (freely available on the Web sites of PLoS Medicine at http://www.plosmedicine.org/, Annals of Internal Medicine at http://www.annals.org/, and Epidemiology at http://www.epidem.com/). Information on the STROBE Initiative is available at http://www.strobe-statement.org.
